# [Retracted] The circadian clock gene *PER2* plays an important role in tumor suppression through regulating tumor-associated genes in human oral squamous cell carcinoma

**DOI:** 10.3892/or.2025.8888

**Published:** 2025-03-19

**Authors:** Xiaoli Su, Dan Chen, Kai Yang, Qin Zhao, Dan Zhao, Xiaoqiang Lv, Yiran Ao

Oncol Rep 38: 472–480, 2017; DOI: 10.3892/or.2017.5653

Following the publication of this article, a concerned reader drew to the attention of the Editorial Office that certain of the tumor images featured in Fig. 6A on p. 478 bore a striking resemblance to tumor images included in three other papers contributed by the same research group, albeit photographed in some cases in slightly different orientations, where the data were intended to show the results from differently performed experiments.

The Editorial Office has conducted a separate investigation of the data in this paper, and concurs with the concerned reader that certain of the tumor images had been re-used in these papers. Owing to the fact that the contentious data in Fig. 6 of the above article were also already under consideration for publication prior to its submission to *Oncology Reports*, or had already been published, the Editor has decided that this paper should be retracted from the Journal. The authors were asked for an explanation to account for these concerns, but the Editorial Office did not receive a reply. The Editor apologizes to the readership for any inconvenience caused.

